# Effect of plasma-induced oxidation on NK cell immune checkpoint ligands: A computational-experimental approach

**DOI:** 10.1016/j.redox.2024.103381

**Published:** 2024-10-01

**Authors:** Pepijn Heirman, Hanne Verswyvel, Mauranne Bauwens, Maksudbek Yusupov, Jorrit De Waele, Abraham Lin, Evelien Smits, Annemie Bogaerts

**Affiliations:** a Research Group PLASMANT, Department of Chemistry, University of Antwerp, 2610, Antwerp, Wilrijk, Belgium; b Center for Oncological Research (CORE), Integrated Personalized and Precision Oncology Network (IPPON), University of Antwerp, 2610, Antwerp, Wilrijk, Belgium; c Institute of Fundamental and Applied Research, National Research University TIIAME, 100000, Tashkent, Uzbekistan; d Laboratory of Experimental Biophysics, Center for Advanced Technologies, 100174, Tashkent, Uzbekistan

**Keywords:** Non-thermal plasma, Natural killer cells, Immune checkpoints, Cancer immunotherapy, Umbrella sampling, Oxidative stress

## Abstract

Non-thermal plasma (NTP) shows promise as a potent anti-cancer therapy with both cytotoxic and immunomodulatory effects. In this study, we investigate the chemical and biological effects of NTP-induced oxidation on several key, determinant immune checkpoints of natural killer (NK) cell function. We used molecular dynamics (MD) and umbrella sampling simulations to investigate the effect of NTP-induced oxidative changes on the MHC-I complexes HLA-Cw4 and HLA-E. Our simulations indicate that these chemical alterations do not significantly affect the binding affinity of these markers to their corresponding NK cell receptor, which is supported with experimental read-outs of ligand expression on human head and neck squamous cell carcinoma cells after NTP application. Broadening our scope to other key ligands for NK cell reactivity, we demonstrate rapid reduction in CD155 and CD112, target ligands of the inhibitory TIGIT axis, and in immune checkpoint CD73 immediately after treatment. Besides these transient chemical alterations, the reactive species in NTP cause a cascade of downstream cellular reactions. This is underlined by the upregulation of the stress proteins MICA/B, potent ligands for NK cell activation, 24 h post treatment. Taken together, this work corroborates the immunomodulatory potential of NTP, and sheds light on the interaction mechanisms between NTP and cancer cells.

## Graphical abstract

**Figure:**
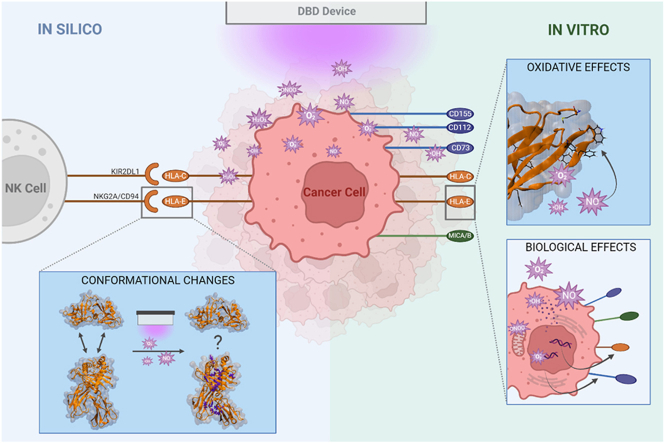

## 1 Introduction

Immunotherapy has emerged as a groundbreaking approach in the fight against cancer, harnessing the body's own immune system to target and destroy cancer cells [1]. Despite their great potential, immunotherapies are not without challenges, which include treatment resistance, variable patient responses, and significant adverse effects [2]. In this light, research continues to explore novel strategies to reinforce therapeutic efficiency while minimizing off-target effects.

The use of non-thermal plasma (NTP) in oncology has been the focus of intense research in recent years [3]. This partially ionized gas generates a complex mixture of reactive oxygen and nitrogen species (RONS) [4,5]. Various RONS are involved in diverse biological processes, including functioning as messenger molecules [6]. In addition, these reactive species play a pivotal role in inflammation and immune cell functioning [[7], [8], [9]]. Indeed, beside the direct cytotoxic effects on cancer cells, the focus in plasma-oncology research lies increasingly on the immunotherapeutic potential of NTP treatment [10,11]. Recent studies have demonstrated that NTP can induce immunogenic cell death [[11], [12], [13], [14]], a cell death mechanism that increases the visibility of the cancer cells to the immune system [15]. In addition, NTP treatment has shown positive effects on various immune cell types: macrophages exhibited an improved response against plasma-treated cancer cells [16,17], while dendritic cells indicated improved infiltration and antigen presentation after treatment *in vivo* [18]. On the other hand, the effect of NTP on other immune cells, such as natural killer (NK) cells, has not yet received much attention [11].

NK cells are lymphocytes of the innate immune system that play a major role in the body's cancer immunosurveillance [19]. Because of their ability to directly recognize and kill cancer cells, they form an attractive target in immunotherapy [20,21]. We have previously reported that treatment of skin cancer cells with NTP augmented NK cell-mediated toxicity *in vitro* [22]. Improved NK cell cytotoxicity was also shown *in vivo*, after treatment of melanoma tumors with NTP [18]. The *in vitro* effects were attributed to the observed change of surface ligand expression on the treated cancer cells. Indeed, NK cell activity relies on the balance between activating and inhibiting signals received through surface receptors, which bind to relevant ligands on the target cell membrane [23]. The interaction between these immune cells and cancer cells is complex, involving numerous ligand-receptor interactions that contribute to the delicate activating/inhibiting balance. Hence, a comprehensive analysis of several key tumor cell-NK cell axes would enhance our understanding of the signaling mechanisms underlying the improved NK cytotoxicity.

Among the key ligands that inhibit NK cells are major histocompatibility complex class 1 (MHC-I) molecules. Unlike antigen-specific T cells, which detect the altered self of cancer cells via intracellular peptides presented by MHC-I, NK cells detect the presence of MHC-I itself. The absence of MHC-I – termed “missing self” – triggers NK cell activation. NK cells express several receptors that recognize MHC-I, with killer immunoglobulin-like receptors (KIR) recognizing the classical HLA-A, B and C ligands, and natural killing group 2, type A, (NKG2A) acting as an inhibitory receptor for the non-classical MHC-I complex HLA-E [[23], [24], [25], [26]].

We previously showed altered expression of both HLA-A/B/C and HLA-E on skin cancer cells after NTP treatment *in vitro* [22]. The fact that NTP treatment is able to change the expression of surface ligands that are important for cancer – immune cell crosstalk is now observed in several studies, and is an appealing therapeutic characteristic for further investigation [18,22,27].

In addition to affecting the expression of ligands on the cancer cell surface, RONS can interact directly with the ligands already expressed on the cells. NTP treatment can induce a broad range of post-translational modifications (PTMs) in proteins [28], which can lead to conformational changes and affect their ability to bind to receptors [29]. To investigate these effects, computational molecular dynamics (MD) simulations have proven a valuable tool [30]. For example, Yusupov et al. [31] showed that oxidation of cell adhesion protein CD44 and its ligand, hyaluronan, significantly decreased their binding affinity, and found indications that the breaking of important disulfide bonds near the binding groove plays an important role. Lin et al. [32] reported that the disruption of salt bridges of immune checkpoint CD47 connecting it to its ligand, caused by oxidation of Lys residues, was associated with conformational changes, and decreased the binding affinity of the CD47 – SIRPα complex. On the other hand, oxidation does not necessarily disrupt normal protein function in all cases. Indeed, while Ghasemitarei et al. [33] found that oxidation of cysteine inside the protein channel of the xC- antiporter severely impaired cysteine uptake by the channel, a similar oxidation did not have a strong effect on transport through the AQP1 channel, as shown by Yusupov et al. [34].

In this study, we employed non-reactive MD simulations to investigate the effect of NTP-induced oxidative changes on the MHC-I complexes HLA-Cw4 and HLA-E. Umbrella sampling (US) was used to determine the binding energy of these MHC-I complexes to their NK cell receptor, respectively KIR2DL1 and NKG2A/CD94. To complement these simulations with experiments, we analyzed the expression of these ligands on three head and neck squamous cell carcinoma (HNSCC) cancer cell lines, a cancer type known for NK cell enrichment [35]. Our data reveals limited effects of therapy-induced oxidation in both the *in silico* and *in vitro* approach. Expanding our focus, we performed flow cytometric analyses on the expression of various NK-regulating ligands, including the T cell immunoreceptor with Ig and ITIM domains (TIGIT) receptor ligands CD155 and CD112 and immune checkpoint CD73, and discovered a rapid reduction of these targets upon NTP exposure. Furthermore, the NK cell activating MICA/B proteins were upregulated on the cellular membrane 24 h post-treatment. Our study unravels appealing targets for therapy-induced oxidation and sheds light on the complex chemical and biological interactions between NTP and cancer cells, with regard to NK cell recognition. This data can provide the groundwork towards rationally designed combination of NTP with exciting and newly developed immune therapies to improve treatment efficiency and accelerate the clinical introduction of this appealing agent.

## 2 Methods

### 2.1 Preparation of the model systems

**Fig. 1:**
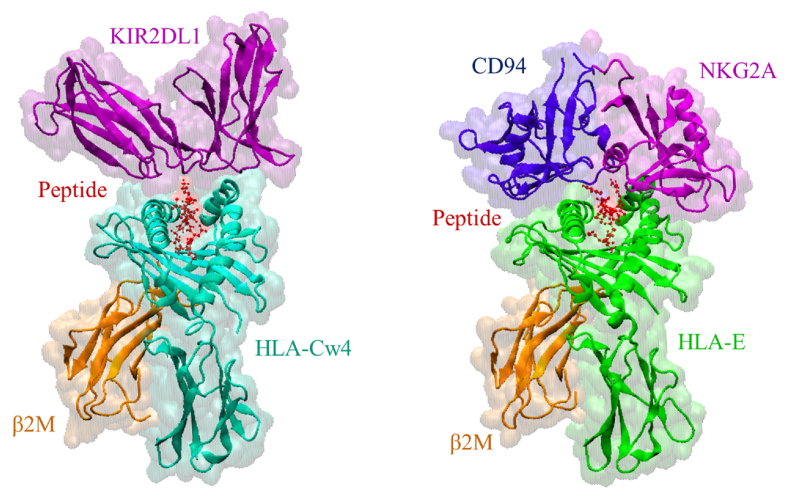
**Schematic representation of the two protein complexes computationally studied**, i.e., HLA-Cw4 – KIR2DL1 (left) and HLA-E − NKG2A/CD94 (right).

Fig. 1 illustrates the two NK cell inhibiting ligand-receptor protein complexes computationally investigated in this study, i.e., HLA-Cw4 – KIR2DL1 (left) and HLA-E − NKG2A/CD94 (right). MHC-I molecules consist of a variable α-chain that presents an intracellular peptide (shown in red), and the smaller β2-microglobulin (β2M, shown in orange) [36]. HLA-E is recognized by the NK cell inhibiting receptor NKG2A, which forms a heterodimer with CD94 in order to become a functional receptor. In contrast to HLA-E, which has a low degree of polymorphism [37], HLA-A, B and C are extremely diverse, as is the group of NK cell receptors that bind to them, i.e. KIR. As discussed by Parham [38], HLA-C is the most important classical MHC-I protein in humans. In addition, of the group of inhibitory KIRs that recognize the various types of HLA-C, KIR2DL1 is the most inhibitory to NK cells.

To elucidate the effect of oxidation on the binding of the investigated complexes, we performed non-reactive MD simulations. The simulations were performed with the GROMACS software (version 2020.2) [39], employing the GROMOS 54A7 force field [40]. The crystal structures of both the NKG2A/CD94 – HLA-E complex and the KIR2DL1 – HLA-Cw4 complex were obtained from the Protein Data Bank (ID: 3CDG [41] and 1IM9 [42], respectively). Force field parameters for the oxidized amino acids were obtained from Ref. [43].

Each model system was prepared by placing the protein complex in a cubic simulation box with periodic boundary conditions applied in all Cartesian directions, and solvating in SPC water [44] containing a concentration of 150 mM NaCl. A minimum distance of 1 nm was ensured between the protein and the box sides to adhere to the minimum image criterion. The system was energy minimized using the steepest descent algorithm, followed by a 2 ns equilibration in the canonical (NVT) ensemble (i.e. a constant number of particles (N), volume (V) and temperature (T)) while applying position restraints with a force constant of 10 000 kJ mol^−1^ nm^−2^ to the heavy atoms of the proteins. Afterwards, a series of equilibrations, totaling 10 ns, was performed in the isothermal-isobaric (NPT) ensemble (i.e. with a constant pressure (P) instead of a constant volume) while enforcing decreasingly strong position restraints (10 000 kJ mol^−1^ nm^−2^ for 2 ns, 1000 kJ mol^−1^ nm^−2^ for 2 ns, 200 kJ mol^−1^ nm^−2^ for 6 ns). In this way, the complexes were gradually equilibrated. The equilibrations were performed at 310 K and 1.0 bar to mimic the conditions the proteins would experience in the body, employing the v-rescale thermostat [45] with a coupling constant of 0.1 ps and the Parrinello-Rahman barostat [46] with a compressibility and coupling constant of 4.5 × 10^−5^ bar^−1^ and 2 ps, respectively. Electrostatics in the system were treated using the reaction field (RF) method [47], while using a cutoff distance of 1.4 nm for the van der Waals and Coulomb interactions. All simulations were performed with a time step of 2 fs. A final equilibration, without enforcing any position restraints on the system, was performed for up to 350 ns, depending on the simulated system.

Each system was simulated in triplicate, with different initial velocities, meaning a total of 12 systems (2 protein complexes, both in native and oxidized states) were used for our investigation. The final equilibration of each system was used to calculate the root-mean-square deviation (RMSD) of the alpha-carbons (Cα atoms) of the protein complexes. In addition, the last 100 ns were used to investigate the root-mean-square fluctuations (RMSF) of the protein residues, as well as the secondary structure of the fully equilibrated complexes and the salt bridge connections between ligand and receptor. For the latter, the VMD software [48] was used.

### 2.2 Investigating the binding affinity of the complexes

To investigate the binding affinity of the complexes, the fully equilibrated protein complex was placed in a new, triclinic simulation box that was elongated in the z-direction, and again solvated in SPC water containing 150 mM NaCl. The complex itself was oriented along the z-axis, i.e., the contact plane between the ligand and receptor was made approximately perpendicular to the z-axis. Next, a new energy minimization, NVT equilibration (2 ns) and NPT equilibration (30 ns) were performed, all while applying position restraints of 1000 kJ mol^−1^ nm^−2^ to the heavy atoms of the protein.

The complex was then pulled apart by subjecting the NK cell receptor (i.e., NKG2A/CD94 or KIR2DL1) to a harmonic potential with a force constant of 1000 kJ mol^−1^ nm^−2^, at a constant velocity of 0.1 nm/ns for 40 ns. The center of mass of NKG2A/CD94 was pulled against the backbone atoms of Lys6, Tyr7, Phe8 and His9 of the HLA-E protein, while the center of mass of KIR2DL1 was pulled against the backbone atoms of Gln96, Arg97, Met98 and Phe99 of the HLA-Cw4. The above residues were chosen for each complex because they are buried inside the protein under the ligand-receptor binding domain, are approximately centered around the z-axis, and exhibit minimal fluctuations compared to other residues in the protein. To prevent movement of the ligand while retaining its flexibility, position restraints were applied to the Cα atoms of Phe36, Cys101, Phe116 and Cys203 (for HLA-E) and Glu63, Tyr118, Tyr159 and Ala205 (for HLA-Cw4). These residues were again chosen as they are buried inside the protein and exhibit low fluctuation. Finally, to prevent movement of the pulled protein (i.e. NKG2A/CD94 or KIR2DL1) in the xy-plane, so-called flat-bottomed position restraints were applied with a radius of 0.05 nm and a force constant of 500 kJ mol^−1^ nm^−2^.

Along the reaction coordinate of each pulling simulation, 40 frames (later supplemented up to 53 frames to ensure adequate sampling) separated by 0.1 nm were isolated to serve as the initial structure in US simulations [49] to sample conformational space in a window at that pulled distance. For the native systems, US was performed for 50 ns, while for the oxidized complexes the US was performed for 75 ns. The last 25 ns of each US simulation were used for data collection (while the first part of the US was used to equilibrate the frame). To extract the free energy profiles along the pulling coordinate, the weighted histogram analysis method (WHAM) was employed [50].

### 2.3 Building the oxidized structures

The oxidized version of both investigated protein complexes was constructed with the Vienna-PTM web server [51] by replacing relevant native amino acids with their oxidized form. Which amino acids would be oxidized by NTP treatment was determined based on two factors: (i) the susceptibility of different amino acids to oxidation, and (ii) the accessibility of these amino acids in the investigated proteins to the solvent, and thus to plasma-produced RONS.

**Fig. 2:**
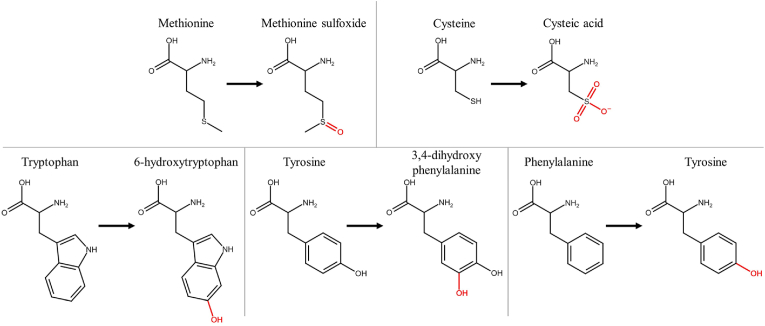
Chemical structures of sulfur-containing (top) and aromatic (bottom) amino acids with their oxidized forms (i.e. structures with atoms in red).

Different amino acids have different susceptibility to PTM after plasma treatment. Takai et al. [52] experimentally investigated the chemical modification of amino acids in solution through treatment with a plasma jet. A similar study was performed by Zhou et al. [53]. Wenske et al. [28,54] reported the PTMs caused by plasma treatment, with both the kINPen and COST-jet, in different peptides, representing a more realistic environment for the different amino acids. It is clear that the amino acids that are most susceptible to plasma-induced PTM are the sulfur-containing methionine and cysteine. They are followed by the aromatic amino acids tryptophan, tyrosine and phenylalanine. Though NTP-induced modifications have been reported for most of the remaining amino acids, they exhibit a far lower susceptibility, e.g. only showing signs of PTM after lengthy NTP-treatment. Which PTM occurs depends on various factors, including the plasma working gas, the plasma device, and the amino acid environment. The most common PTM induced by plasma, however, seems to be oxidation of the amino acid side chains [28]. Based on this, Fig. 2 shows the plasma-induced PTMs we considered in our investigation.

To determine which of these amino acids specifically in our investigated complexes would be available for oxidation by plasma treatment, we analyzed the solvent accessible surface area (SASA) of the proteins. For this, we repeated all simulation steps outlined in section 2.1 for only the ligands of the complexes, i.e. HLA-E and HLA-Cw4. Indeed, in an experimental setting, plasma treatment of cancer cells would cause oxidation of only the ligands expressed on the cancer cells. The last 20 ns of the equilibrations were used for the SASA analysis. By comparing the SASA to the hydrophilic surface area of the amino acids in question [55], we can determine which amino acids can be easily reached by the solvent, and thus, by plasma-produced RONS.

### 2.4 Cell culture

Three human head and neck squamous cell carcinoma cell lines, Cal27, SCC61 and SCC22B, were used in this study. The Cal27 cell line was obtained from American Type Culture Collection (ATCC, Rockville, MD, USA), the SCC61 cell line was kindly provided by Prof. Dr. Sandra Nuyts (University Hospital Leuven, Leuven, Belgium), and SCC22B cells were kindly provided by Prof. Dr. Olivier De Wever (Laboratory of Experimental Cancer Research, Ghent University Hospital, Ghent, Belgium). Cell lines were commercially available and no ethical approval was required. Cells were cultured in Dulbecco's Modified Eagle Medium (DMEM, Life Technologies), supplemented with 10 % fetal bovine serum (FBS, Life Technologies), 1 % l-glutamine (Life Technologies), and 1 % Penicillin/Streptomycin (P/S; Life Technologies). Cell cultures were incubated in a humidified atmosphere at 37 °C and 5 % CO2 conditions, and passaged when 80 % confluence was reached to maintain exponential growth. Cell identity was confirmed via short tandem repeat profiling, and regular checks for mycoplasma infection were performed.

### 2.5 NTP treatment

A microsecond-pulsed dielectric barrier discharge (DBD) system, described in our previous work [18,32,56,57], was used for the experimental part of this work. The set-up comprises a microsecond-pulsed power supply (Megaimpulse Ltd, Russia), with a round-bottom dielectrically covered electrode, providing an electrically and thermally stable plasma [18]. Detailed operation parameters can be found in Table 1.

**Table 1:** Experimental parameters for DBD-NTP application.

Electrical and Operating Parameters	
Pulse voltage amplitude	30 kV
Pulse rise time	1–1.5 μs
Pulse width	2 μs
Pulse frequency	200 and 500 Hz
Treatment time	10 s
Application distance	1 mm

For all experiments, this microsecond-pulsed DBD system was used to treat HNSCC cells in 24-well plates when a confluence of 80 % was reached, one day after experimental seeding. Cell culture medium was removed immediately prior to NTP application and the 24-well DBD probe was lowered into the well to treatment distance (1 mm), using a z-positioner. Right after treatment, cells were overlaid with fresh culture medium. Plates were incubated for a day at 37 °C and 5 % CO_2_ for 24 h post-treatment analysis, or processed right after treatment for immediate (0 h) analysis.

### 2.6 Flow cytometry analysis of NK ligands on HNSCC tumor cells

The NK ligands included in this study were measured individually via a dual staining of a viability stain (LIVE/DEAD™ Fixable Near-IR, APC-Cy7), and a monoclonal antibody against the target ligand (PE *anti*-HLA-C, *anti*-HLA-E, anti-CD155, anti-CD122, anti-CD73, or anti-MICA/B). Ligand expression was measured at two distinct time points to establish a clear temporal separation between immediate chemical and later biological effects at 24 h. In short, cells were washed with phosphate buffer saline (PBS), detached with accutase (Sigma-Aldrich), spun down and resuspended in FACS buffer (sheath buffer (BD Biosciences), with 0.1 % BSA and 0.05 % NaN_3_). Cell density of untreated controls and NTP-treated samples was set at 5 × 10^5^ cells/ml. Samples were incubated with 1 μL of LIVE/DEAD™ Fixable Near-IR (L10119, ThermoFisher Scientific), and a single stain for a target ligand: *anti*-HLA-C (5 μL, Cat. No. 566372, BD), *anti*-HLA-E (5 μL, 12-9953-42, Invitrogen), anti-CD155 (5 μL, 566718, BD), anti-CD112 (5 μL, 337410, BioLegend), anti-CD73 (5 μL, 12-0739-42, Invitrogen), or anti-MICA/B (20 μL, 558352, BD). Fluorescence Minus One (FMO) gating controls were included for all cell lines and treatment conditions. After 30 min of incubation in the dark at 4 °C, cells were washed twice with FACS buffer, and resuspended in 100 μL FACS buffer for read-outs. Sample acquisition was performed on the NovoCyte Quanteon (Agilent Technologies). Experimental data was gated and analyzed using the FlowJo software version 10.8.1 (FlowJo LLC, Ashland, OR, USA) (Supplementary Fig. 1).

### 2.7 Statistical analysis

Prior to statistical calculations, the Grubbs' Test was performed to detect significant outliers in the experimental data. Afterwards, the linear mixed model in JMP Pro 17 (SAS Software, Tervuren, Belgium) was used to analyze statistical differences between treatment conditions. NTP exposure was designated as the fixed effect, while the different experimental repeats and the interaction between experiments and the date they were performed were considered as random effects. The random slope model was only retained when the treatment-date interaction was significant (p ≤ 0.05). Statistical difference by the fixed effect was determined, and the post-hoc Dunnett's test was used to calculate adjusted p values of the treatments compared to untreated controls. P values equal or less than 0.05 were considered as statistically significant. Data in the experimental graphs is represented as mean ± SEM, with individual values shown as dots in the bar plots.

## 3 Results

### 3.1 Oxidation affects the protein complex structure, but not the binding free energy

To gain insight into how NTP-induced oxidation affects the MHC-I complexes HLA-Cw4 and HLA-E, we performed non-reactive MD simulations for both the native and oxidized version of both protein complexes. To construct the oxidized versions of the ligands, we chose to implement the oxidation products of only the amino acids that are known to be most susceptible to NTP-oxidation, i.e. Met, Cys, Trp, Tyr, and Phe, that additionally have a high solvent accessibility in the simulated proteins, meaning they can be reached by RONS dissolved in the surrounding liquid during NTP treatment. Table 2 indicates which amino acids were oxidized in our simulated systems. Notably, all cysteine residues in both HLA-Cw4 and HLA-E have a very low solvent accessibility, thus preventing oxidation of the disulfide bonds present in the protein complexes.

**Table 2:** List of amino acids used to construct the oxidized forms of the ligands (i.e. HLA-Cw4 and HLA-E).

	KIR2DL1 – HLA-Cw4	NKG2A/CD94 – HLA-E
**HLA**	Met98, Trp14, Trp133, Trp147, Trp204, Tyr84, Tyr159, Phe8, Phe99, Phe116, Phe241	Met98, Trp51, Trp60, Trp204, Trp244, Tyr84, Tyr159, Phe8, Phe109, Phe116
**β2M**	Met0, Trp60, Tyr10, Tyr63, Phe22, Phe56, Phe62	Met0, Trp60, Tyr10, Tyr63, Phe22, Phe56, Phe62
**peptide**	/	Met2

**Fig. 3:**
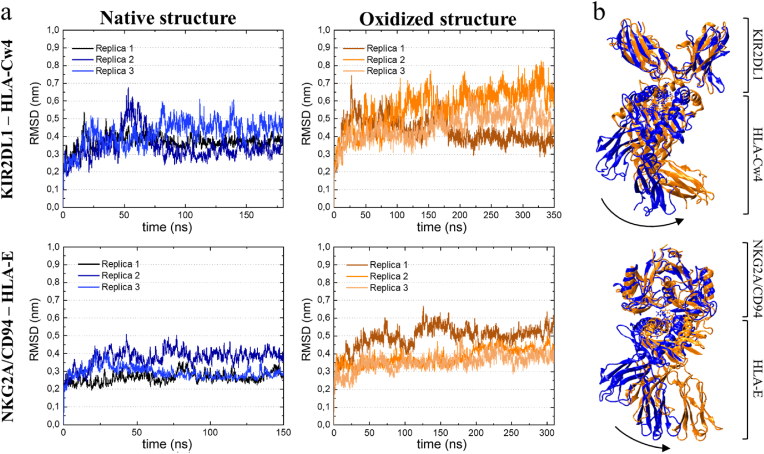
**Oxidation-induced structural changes in the proteins.** (a) RMSD of the Cα atoms of the native and oxidized protein complexes (b) Cartoon view of representative frames (i.e., one of their replicas). for the native (blue) and oxidized (orange) protein complexes, aligned based on the non-oxidized receptors. The arrows highlight how the ligands have shifted in the oxidized structure.

**Fig. 4:**
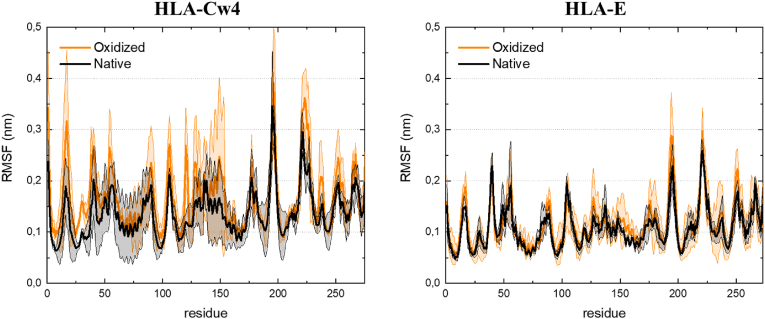
**Protein flexibility is not significantly changed in the oxidized structures.** The graphs show the RMSF of the residues of the native and oxidized ligands (i.e. HLA-Cw4 and HLA-E), averaged over the three replicas for each structure. Errors associated with sampling are shaded accordingly.

Fig. 3a shows the calculated RMSD of the simulated protein complexes in both native and oxidized form. Protein oxidation has been reported to cause conformational changes and to result in higher protein flexibility [30], as evident from larger (fluctuations in) RMSD. The oxidized versions of the simulated MHC-I complexes converge to an RMSD that is on average higher than the native structures. For HLA-Cw4 – KIR2DL1, the RMSD of the oxidized structure converges to an average value of 0.52 nm (averaged over the three replicas), compared to 0.38 nm for the native structure, while for HLA-E − NKG2A/CD94, the average converged RMSD is 0.44 nm for the oxidized structure, compared to 0.32 nm for the native structure, indicating that the oxidized proteins indeed equilibrated to a different conformation. On the other hand, the RMSD fluctuations do not exhibit a clear difference, which indicates that the flexibility of the ligands is not affected to a large degree by the oxidative changes. This is supported by the calculated RMSF of the ligands, as shown in Fig. 4. For HLA-E, the RMSF is nearly unchanged after oxidation. For HLA-Cw4, the RMSF of the oxidized system is slightly higher in some regions, particularly near the oxidized residues, indicating a slightly increased local flexibility. Notably, the native HLA-Cw4 was calculated to already be more flexible than HLA-E.

While the RMSD of both simulated MHC-I complexes suggests they undergo a change in conformation after NTP-induced oxidation, they almost completely retain their secondary structure. Analysis of the secondary structure of the ligands is shown in Table 3. In the case of HLA-Cw4, the most prominent alteration in the secondary structure is a decrease in the turn structure, accompanied by an increase in random coil structures. For HLA-E, the most notable change in secondary structure is a slight decrease in the helical structure, again accompanied by an increase of random coils. Visual inspection indicated that this change is caused by denaturation of the helix between residues 49 and 54, likely as a result of the two tryptophan oxidations (Trp51 and Trp60) in this region. Visual inspection also revealed that the most notable conformational change of the oxidized proteins compared to their native structure is a pivot of the ligand with respect to the domain that binds to the receptor. This occurs for both simulated protein complexes, and explains the higher average RMSD, although the precise extent of the pivot differed over time (during the simulation, as the protein conformation fluctuates) and between the simulation replicas. The pivot is clearest when aligning the native and oxidized version of the protein based on only the receptor chains, i.e. the protein chains that were not oxidized, which is shown in Fig. 3b.

**Table 3:** Secondary structure analysis of the native and oxidized ligands (i.e. HLA-Cw4 and HLA-E). The values given indicate the relative occurrence (in %) of different conformations.

	HLA-Cw4		HLA-E	
	Native	Oxidized	Native	Oxidized
Turn	25.2 ± 1.2	22.1 ± 1.2	23.7 ± 1.2	23.4 ± 1.2
β-sheet/β-bridge	41.0 ± 1.0	39.8 ± 1.1	41.8 ± 0.8	41.1 ± 0.8
Helix	17.6 ± 0.8	18.4 ± 0.8	18.2 ± 0.7	17.7 ± 0.6
Random coil	16.2 ± 1.2	19.7 ± 1.3	16.3 ± 1.0	17.8 ± 1.0

**Fig. 5:**
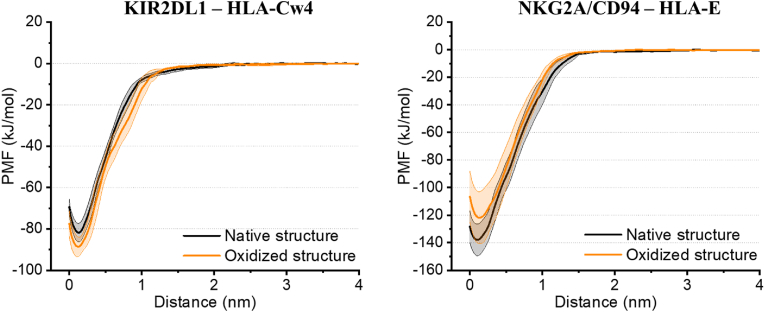
**Oxidation does not affect the binding affinity of the protein complexes.** Free energy profiles (i.e. potential of mean force, PMF) of the native and oxidized protein complexes. Errors associated with sampling are shaded accordingly.

To investigate if the oxidation affects the binding affinity of the investigated protein complexes, we performed pulling simulations followed by US simulations to determine the free energy profiles along the binding coordinate for each complex. One can see in Fig. 5 that the oxidation of the ligands had minimal effect on the profiles. The binding free energy, i.e. the depth of the potential well, was calculated to be −82 ± 4 kJ/mol for HLA-Cw4 – KIR2DL1 in its native form, changing to −89 ± 5 kJ/mol when oxidized. For the HLA-E − NKG2A/CD94 complex, the calculated binding energies of the native and oxidized forms are −138 ± 12 kJ/mol and −122 ± 19 kJ/mol, respectively. Our simulations thus predict that, given the errors associated with the energy profiles, both investigated ligands respectively have similar binding affinity to their NK cell receptor regardless of being subjected to oxidation through plasma treatment.

### 3.2 A closer look at the connections between the ligands and receptors

The binding of HLA-E to NKG2A/CD94 is dominated by polar interactions mediated by several hydrogen bonds and a number of salt bridges. The crystal structure of HLA-E − NKG2A/CD94 exhibits five salt bridges [41]. Table 4 shows the binding persistence for the salt bridges found in our simulations, i.e. the percentage of time in which the amino acids that form the salt bridge are actually interacting. To consider a salt bridge as “interacting”, we employed a distance threshold of 4.5 Å [58]. In addition, we only included interactions with a binding persistence of over 5 %, unless specified otherwise. The salt bridges that are present in the crystal structure are highlighted in Table 4. Notably, two of these salt bridges (Asp163^CD94^ – Arg75^HLA−E^ and Arg137^NKG2A^ – Glu154^HLA−E^) had a very low binding persistence in our simulations. On the other hand, several salt bridges not reported to be present in the crystal structure are predicted by our simulations to be (transiently) present in solvated state.

**Table 4:** Binding persistence (i.e. percentage of time that the N-O distances are below 4.5 Å) of the salt bridges that play an important role in the ligand-receptor complex systems, during the last 100 ns of the three replicate simulations. The salt bridges that are present in the crystal structure of the complexes are written in italic.

		Salt bridge binding	
HLA-E	CD94	Native	Oxidized
Arg68	Glu164	29 %	20 %
Arg68	Asp168	51 %	30 %
***Asp69***	***Arg171***	***26 %***	***20 %***
***Arg75***	***Asp163***	***1 %***	***4 %***
Arg75	Glu164	9 %	4 %
Arg79	Asp163	26 %	19 %

**HLA-E**	**NKG2A**		
Asp149	Arg137	8 %	5 %
***Glu154***	***Arg137***	***6 %***	***7 %***
Glu161	Lys217	28 %	8 %
***Asp162***	***Lys199***	***37 %***	***47 %***
Asp162	Arg215	17 %	6 %
***Asp162***	***Lys217***	***15 %***	***20 %***
Glu166	Lys199	4 %	34 %

		Salt bridge binding	
HLA-Cw4	KIR2DL1	Native	Oxidized
Arg69	Glu21	12 %	0 %
Lys80	Asp183	42 %	25 %
***Lys80***	***Glu187***	***47 %***	***68 %***
***Arg145***	***Asp135***	***18 %***	***5 %***
Lys146	Asp135	0 %	31 %
***Lys146***	***Asp183***	***90 %***	***2 %***
Lys146	Glu187	15 %	10 %
Lys8 (peptide)	Glu187	30 %	30 %

For the interaction between HLA-E and CD94, the most notable change in the oxidized state is a weaker binding of the salt bridge Arg168^CD94^ – Asp68^HLA−E^, as evidenced by the 21 % decrease in binding persistence. Some of the salt bridges that contribute to the interaction between HLA-E and NKG2A seemingly exhibit a decrease in binding for the oxidized state as well, though this is mostly accompanied by an increase in binding to another amino acid close by. The reduced binding of Asp162^HLA−E^ to Arg215^NKG2A^ is accompanied by an enhanced binding to Lys199^NKG2A^, while the lower binding of Lys217^NKG2A^ to Glu161^HLA−E^ coincides with increased binding to Asp162^HLA−E^. Finally, the binding of salt bridge Lys199^NKG2A^ – Glu166^HLA−E^ is increased for the oxidized complex. However, unlike for the other salt bridges, this changed behavior was present in only one simulation replicate in which this interaction was very significant, while not being significantly present (<10 %) in any of the other replicates.

Compared to NKG2A/CD94, KIR receptors have a smaller interaction surface with MHC-I molecules. This is accompanied by fewer interactions between the receptor and its ligand, which is reflected in the lower calculated binding energy (Fig. 5). The interaction between HLA-Cw4 and KIR2DL1 is again dominated by polar interactions, including three salt bridges that are found in the crystal structure of the complex [42]. Two of these salt bridges (Asp135^KIR2DL1^ – Arg145^HLA−Cw4^ and Asp183^KIR2DL1^ – Lys146^HLA−Cw4^) are conserved across HLA-C molecules, while the third (Glu187^KIR2DL1^ – Lys80^HLA−Cw4^) is unique to KIR2DL1, and plays an especially important role in the binding of this complex [38]. As shown in Table 4, the oxidation of HLA-Cw4 was found to affect these salt bridges in different ways. The Glu187^KIR2DL1^ – Lys80^HLA−Cw4^ salt bridge was calculated to have an increased binding persistence, implying a stronger interaction. Meanwhile, the binding of the other two salt bridges decreases. Both distance changes correspond logically to the observed pivot of oxidized HLA-Cw4 with respect to KIR2DL1, shown earlier in Fig. 3. When looking at the calculated binding free energy, these effects (i.e. the stronger Glu187^KIR2DL1^ – Lys80^HLA−Cw4^ interaction and weaker Asp135^KIR2DL1^ – Arg145^HLA−Cw4^/Asp183^KIR2DL1^ – Lys146^HLA−Cw4^ interaction) however seem to cancel out, as we observed no significant effect of the investigated oxidations on the binding free energy. In addition to the salt bridges discussed above, a few additional salt bridges were found to be transiently present in our simulations. Except for the salt bridge Glu21^KIR2DL1^ – Arg69^HLA−Cw4^, these additional salt bridges are the result of promiscuous interactions between the amino acids that form salt bridges in the crystal structure. A special case is the salt bridge Asp183^KIR2DL1^ – Lys146^HLA−Cw4^. These amino acids did not interact in any of the simulations, as their relative distance was too large, except in replicate 2 of the oxidized system, where a salt bridge formed with a persistence of 92 % in the equilibrated part of the simulation. This replicate underwent the largest pivot of HLA-Cw4 with respect to KIR2DL1, also evident from its RMSD (see Fig. 3a), allowing formation of this new interaction.

In summary, our simulations indicate that NTP-induced oxidations alter the structure of the investigated ligand-receptor complexes, but do not affect their binding strength. To complement these computational results experimentally, we monitored the expression status of these protein complexes following plasma treatment of cancer cells *in vitro*.

### 3.3 MHC-I complexes HLA-C and HLA-E are moderately altered after NTP application

**Fig. 6:**
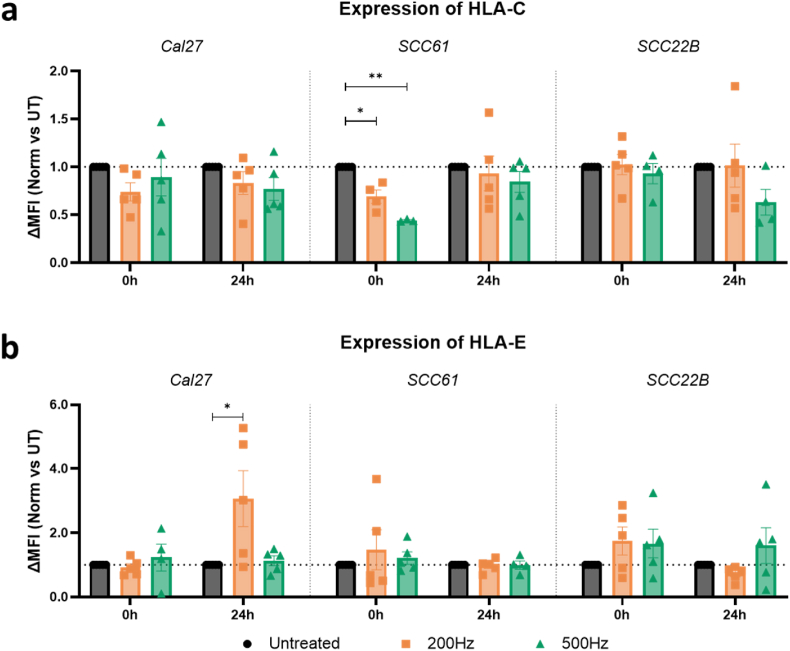
**Exposure to NTP results in moderate expressional changes of the MHC-I complexes HLA-C and HLA-E in several HNSCC cell lines.** Quantification of (a) HLA-C and (b) HLA-E expression after exposure to different regimes of NTP. Results are depicted as mean fluorescence intensity (MFI) minus FMO control, normalized to untreated controls (normalized (Norm) ΔMFI), immediate (0 h) and one day (24 h) post-treatment for all three HNSCC cell lines. Data are represented as mean ± SEM, with individual values shown (n = 5). Statistical significance between untreated cells and the treated conditions was determined using the generalized linear mixed model with post hoc Dunnett's test (∗p ≤ 0.05; ∗∗p ≤ 0.01). Outliers were calculated with the Grubbs' Test.

To determine the immediate oxidative effects of NTP on the MHC-I complex molecules HLA-C and HLA-E in an experimental setting, different HNSCC cell lines were exposed to NTP and immediately analyzed for ligand expression (0 h analysis). While no significant differences in mean fluorescence intensity (normalized ΔMFI) were reported for HLA-E across all cell lines (Fig. 6b), detection of HLA-C decreased significantly compared to untreated controls in the SCC61 cell line (Fig. 6a, middle panel). At an NTP regime of 200 Hz, HLA-C expression was reduced by 1.4-fold, which further diminished to more than a 2-fold reduction in expression levels with a 500 Hz regime (0.69 and 0.44, respectively; *p ≤ 0.0317*).

Besides immediate oxidative effects at the cell surface, NTP exerts more pleiotropic and downstream effects on cancer cells [18,31,59,60]. Therefore, the expression levels of these MHC-I molecules were also measured 24 h after NTP application. This time point was selected with the specific aim of differentiating between the immediate chemical effects (e.g., oxidative) of plasma treatment and the subsequent biological response of the cells at 24 h. Indeed, including an intermediate time point (e.g., 4 or 12 h) would complicate this distinction, as it would likely reflect a mixture of diminishing chemical, but also early biological responses, making it challenging to delineate the independent contributions of each. Cellular responses in HLA-C and HLA-E expression toward NTP were limited for all cell lines, except for a significant upregulation in HLA-E expression (3.07; *p = 0*.*0455*) at a low NTP regimen in the Cal27 cell line (Fig. 6b, left panel).

### 3.4 Key targets in the TIGIT axis are downregulated by NTP-induced ligand oxidation

**Fig. 7:**
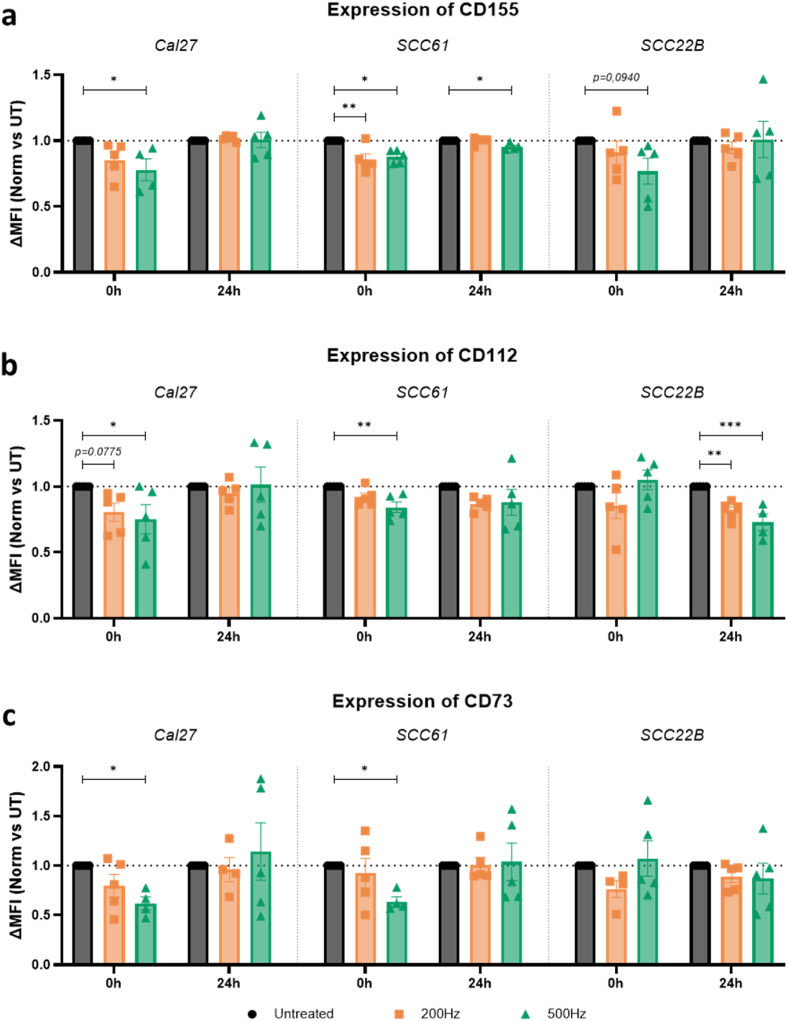
**NTP reduces the expression of key targets in the TIGIT axis and CD73 immediately after treatment exposure**. Flow cytometric quantification of the TIGIT receptor ligands (a) CD155 and (b) CD112, and (c) immune checkpoint CD73. Expression levels are depicted as mean fluorescence intensity minus FMO control, normalized to untreated controls (Norm ΔMFI), immediate (0 h) and one day (24 h) post-treatment for all three HNSCC cell lines. Data are represented as mean ± SEM, with individual values shown (n = 5). Statistical significance between untreated cells and the treated conditions was determined using the generalized linear mixed model with post hoc Dunnett's test (∗p ≤ 0.05; ∗∗p ≤ 0.01; ∗∗∗p ≤ 0.001). Outliers were calculated with the Grubbs' Test.

As NK functionality is determined by the integration of complex activation and inhibition signals rather than a single interaction impulse [[61], [62], [63]], we subsequently investigated a panel of key tumor ligands of the TIGIT receptor, a major inhibitory pathway for NK functionality [61,64]. CD155 and CD112 are inhibition signals for this highly immunosuppressive axis, though CD115 is the dominant antigen while the binding with CD112 is weaker [64,65]. As recent studies indicated that targeting of CD155 and CD73 works synergistically and surpasses the failure in overcoming NK cell dysfunction by CD155 monotherapy [64], we also included this immune checkpoint in the analysis (Fig. 7).

Immediate NTP-induced oxidation of the target ligand resulted in significantly reduced expression of both CD155 and CD112, in the Cal27 and SCC61 cell line with the higher treatment regime (0 h; 500 Hz), as shown in Fig. 7a and b. Both ligands were diminished with 21.9 % and 24.8 %, respectively. For the Cal27 cell line, while 12–15 % reduction was noted for SCC61 (*p ≤ 0.0304*). These effects became even more pronounced for the immune checkpoint CD73 with a strong decline in ΔMFI, around 36 %, for both cell lines (Fig. 7c). It needs to be stated that these oxidative effects are transient, as 24 h later, expression levels stabilized again to baseline. It is apparent that both cell lines responded very similar to therapeutic oxidation, while the SCC22B cell line exploits a more robust, oxidation-resistant phenotype. Although a trend in decreased CD155-ΔMFI could be observed (1 vs 0,77; *p = 0*.*094*), this was not captured in the immediate CD122 and CD73 expression profile (Fig. 7a–c, right panel). Nevertheless, a dose-dependent decrease in CD112 was observed 24 h after treatment, thus suggesting a downstream, stress-induced cellular response in target downregulation, rather than instant oxidative effects of the applied NTP in this cell line.

### 3.5 NTP stimulates the expression of the activating NK ligand MICA/B

**Fig. 8:**
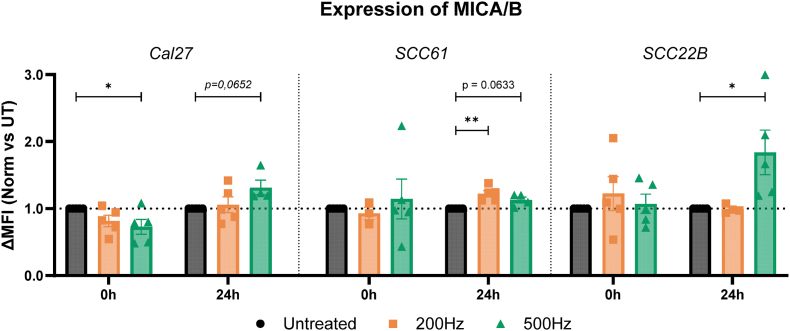
**NTP stimulates the expression of the activating NK ligand MICA/B 24h post NTP treatment.** HNSCC cell lines were treated with different regimes of NTP, and expression of the MICA/B ligands was analyzed with flow cytometry. The amount of MICA/B expression on the cell surface is represented as mean fluorescence intensity minus FMO control, normalized to untreated controls (normalized (Norm) ΔMFI), immediate (0 h) and one day (24 h) post-treatment. Data are represented as mean ± SEM, with individual values shown (n = 5). Statistical significance between untreated cells and the treated conditions was determined using the generalized linear mixed model with post hoc Dunnett's test (∗p ≤ 0.05; ∗∗p ≤ 0.01). Outliers were calculated with the Grubbs' Test.

Lastly, to further evaluate the delicate balance between activating/inhibiting signals on NK cells following NTP treatment, we evaluated the influence of NTP on the activating NK ligand MICA/B. Our data showed that oxidation-induced effects, immediately after application, were limited (Fig. 8). Ligand expression decreased by 28 % (1 vs 0.72; *p = 0.0415*) after 500 Hz application in the Cal27 cell line, while these effects were not measured in the other two cell lines.

Since MICA/B proteins are recognized as stress proteins, induced in tumor cells encountering DNA damage [66], we hypothesized augmentation of ligand expression profile in the different HNSCC cell lines one day post NTP exposure. Indeed, NTP treatment effects mostly manifested in 24 h, with an upregulation of MICA/B on the cellular membrane of all three cell lines compared to untreated controls. Once again, this trend was more pronounced for the higher treatment regimen. Interestingly, the SCC22B cell line, which exhibited the least expressional changes for the inhibitory ligand panel, was strongly positive for MICA/B expression with an 84 % increase in ΔMFI compared to baseline (1 vs 1.84; *p = 0*.*0297*), as displayed in the right panel of Fig. 8.

## 4 Discussion

In this study, we investigated the effects of NTP on different tumor-associated immune checkpoint ligands for NK cells. We used computational methods to gain insight into the effect of NTP-induced chemical changes on MHC Class I molecules HLA-Cw4 and HLA-E, and how the resulting conformational changes affect biological properties like receptor-ligand binding affinity. Furthermore, we experimentally investigated the oxidation-induced effects of NTP on these cell surface molecules, and broadened our scope to other key immune checkpoints affecting NK cell functioning, providing valuable information for subsequent immunological studies. We distinguished between immediate oxidation effects and downstream cellular responses in the cancer cells. Immediate modifications (0 h analysis) to expression profiles were attributed to oxidation-induced effects, as literature delineates this as the most proficient chemical modification [28,31,32,67]. Besides these transient chemical alterations, the reactive species in NTP cause a cascade of downstream cellular reactions, including metabolic alterations, dysregulation of the anti-oxidant system, and changes in genetic and phenotypic expression profiles (24 h analysis) [14,56,68,69].

Employing MD simulations and umbrella sampling, we aimed to investigate the interaction of MHC-I proteins HLA-Cw4 and HLA-E with their receptors, respectively KIR2DL1 and NKG2A/CD94, and the effect of NTP-induced oxidative changes thereon. The interaction of both is dominated by polar interactions. Several salt bridges were found to be present in our simulations apart from those reported to be present in the crystal structure of the proteins. Notably, for HLA-E, the most prominent salt bridge (Arg168^CD94^ – Asp68^HLA−E^) is not among those present in the crystal structure, although it was previously indicated to be important in other condensed-phase simulations [70]. Meanwhile, some salt bridges important in the crystal structure were not prominent in our simulations, either due to different interactions taking the upper hand for the participating amino acids (Asp163^CD94^ – Arg75^HLA−E^), or due to the amino acids simply not interacting much when allowed to move over time (Arg137^NKG2A^ – Glu154^HLA−E^). In the latter case, indeed, it was found experimentally that prohibiting the formation of this salt bridge through mutation did not affect protein binding [71]. For HLA-Cw4, most salt bridges present in our simulation resulted from promiscuous interactions between the amino acids that form salt bridges in the crystal structure.

Oxidation of the protein ligands did not significantly affect their stability in the simulations. The secondary structure was almost completely retained, and the oxidations did not impact the flexibility of the protein. It has previously been reported that plasma-induced oxidations can affect the protein structure, or even induce partial denaturation. These effects were however mostly observed for (small) proteins with solvent-accessible disulfide bridges [29,31,72]. Both MHC-I complexes contain disulfide bridges, important for structural stability, but as these are buried inside the protein, they are unavailable to breakage by NTP-induced oxidation. Although the oxidations do cause the ligands to pivot with respect to their receptor (see Fig. 3b), the binding domain itself remains nearly unaffected, and the calculated binding free energy of the complexes did not change significantly after oxidation. Indeed, most of the solvent accessible amino acids prone to oxidation in the protein complex were found to be located in the α3 region of both HLA-Cw4 and HLA-E, and in β2M. Furthermore, of the amino acids likely oxidized by plasma treatment that are situated in the binding domain of the complex, none play a direct role in the actual interaction between the HLA proteins and their receptors.

In accordance with our computational results, we experimentally observed only minor changes in the expression status of HLA-C and HLA-E by NTP oxidation. A significant decrease in the detection of HLA-C directly after treatment was solely observed in the SCC61 cell line (Fig. 6). In contrast, NTP caused a rapid reduction of the two TIGIT ligands CD155 and CD112, and immune checkpoint CD73 in two out of three tested cell lines (Fig. 7). It is clear that some immune checkpoints are more vulnerable to therapeutic oxidation, and linking this vulnerability to the structure of these proteins forms an interesting avenue for future computational analysis. Even so, it is essential to recognize some nuances in this context, because the experimental approach of binding a monoclonal antibody for fluorescence staining to the target ligand only partially approximates the complete ligand-receptor interaction *in vivo*. Indeed, the binding mechanism of the antibody can be [32], but is not necessarily, the same as that of the biological receptor. Still, far-leading structural changes due to (NTP-induced) oxidation would affect the binding to both antibody and receptor.

24 h after NTP application, the ΔMFI of HLA-C and HLA-E remained around baseline (Fig. 6). The same is true for ligands CD155, CD112, and CD73, indicating that the effects observed immediately after treatment were transient. However, NTP did stimulate the cellular upregulation of the activating NK ligands MICA/B (Fig. 8). This aligns with previous reports, highlighting MICA/B as important stress proteins in response to cellular imbalance and DNA damage, which are key mechanisms of action of NTP [59,73]. Moreover, we can conclude that the NTP investigated in this study modulated expression levels rather than the absolute positivity of tumor cells, as indicated by the Overton analysis. (Supplementary Fig. 2). Apart from MICA/B, other stress-induced proteins are also known to contribute to NK cell activation, such as ULBPs [26] and membrane-bound HSP70 [74,75]. Investigating the effect of NTP treatment on the expression of such stress-induced membrane proteins would further expand our understanding of how NTP can modulate the recognition of cancer cells by NK cells, and will form the subject of future work. In addition, while oxidative modifications likely play a role in later biological observation as well, it is expected that a complex network of biological mechanisms takes place over time, moving from the immediate chemical character to 24 h post-treatment, such as protein degradation (e.g., via proteasomal pathways) and alterations in protein trafficking to and from the cell membrane [31,32,76]. Furthermore, oxidative stress can trigger signaling pathways that regulate the stability and localization of membrane proteins. NTP treatment can also modulate gene expression, leading to changes in the synthesis of key proteins that disrupt membrane lipid organization and impact the localization of proteins associated with lipid rafts [18,56]. Deeper exploration of these underlying pathways can be built on the work we presented here. Consequently, a detailed temporal analysis of ligand expression over a series of time points would provide valuable insights into the kinetic profile and dynamics of these NTP's pleiotropic effects.

It is apparent that sensitivity to NTP and the resulting outcome varied between the studied cell lines. While Cal27 and SCC61 were derived from tongue lesions [77,78], the SCC22B cell line, originated from a metastatic lymph node in the neck [78]. Interestingly, the expression profiles of Cal27 and SCC61 cells clustered together, whereas the SCC22B cells exhibited a markedly distinct therapeutic response, particularly for the expression of CD112 and CD73. To underline this, we repeated the analysis for CD47, an innate immune cell checkpoint which was previously studied in our lab [32], for the cell lines investigated in the present paper. Indeed, NTP treatment modulated CD47 expression differently across the cell lines (Supplementary Fig. 3). The fact that different behaviors can be seen immediately after treatment indicates that biological effects beyond purely the chemical protein alterations are at play. Further studies investigating underlying mechanisms (e.g., genetic profile, tumor subsite, disease stage) are necessary to fully elucidate the implications and biological relevance of these observations. Furthermore, in-depth analysis of the chemical alterations in protein structure using mass spectrometry could be a valuable subsequent step to shed light on these observations, and potentially unravel additional post-translational modifications relevant for instant ligand reduction (e.g. nitrosylation).

The results presented in this work build upon previous work performed in our lab combining experimental and computational methods [31,32] and broaden our knowledge on crucial aspects of the interaction between NTP, cancer cells, and immune cells. Indeed, our research on the cytotoxic capacities of NK cells following NTP application has previously demonstrated enhanced NK cell-mediated killing in both *in vitro* and *in vivo* melanoma models [18,22]. The objective of this manuscript was to build further on these functional observations, while specifically highlighting the effects of NTP on key tumor cell-NK cell signaling axes. Our results demonstrate a significant impact of NTP application on different inhibitory and activating ligands and pathways, critical for NK cell functioning, but also indicate that these effects are dependent on both the specific protein and cell type. This data corroborates the robust immunomodulatory capabilities of NTP. Specifically, it broadens the understanding that NTP not only enhances tumor immunogenicity through the induction of immunogenic cancer cell death [14,68], but also encompasses the modulation of crucial immune checkpoints within the tumor microenvironment. Hence, the compelling character of the highly reactive yet localized treatment profile of the complex mixture of species in NTP may render it an attractive candidate as a combination partner to bolster existing immune therapies facing critical challenges such as ligand shedding and immune evasion [66,79,80]. Nevertheless, further research is needed to fully understand the clinical relevance of our work, and to further investigate the effects of NTP on NK cell-mediated cytotoxicity, for both HNSCC as well as other cancer types. Additionally, within the context of this research scope, it would be intriguing to investigate the impact of oxidation-induced treatment effects on well-established immunological targets such as the PD-1/PD-L1 axis.

## 5 Conclusion

Using both *in silico* and *in vitro* methods, we investigated the effects of NTP treatment on different tumor-associated immune checkpoint ligands for NK cells. Taken together, our computational results indicate that MHC Class I molecules HLA-Cw4 and HLA-E are not significantly affected by NTP-induced oxidative changes. Although the structure of the ligand-receptor complexes was altered, this did not result in a changed binding strength. Accordingly, we experimentally observed only minor alterations in the expression status of HLA-C and HLA-E, both immediately after treatment and 24 h later. In contrast, NTP caused a rapid reduction of the two TIGIT ligands CD155 and CD112, and immune checkpoint CD73. Meanwhile, the well-known stress proteins and activating NK cell ligands MICA/B were upregulated 24 h after treatment. Taken together, our results demonstrate that NTP affects different ligands important for NK cell functioning, but also that these effects are dependent on both the specific protein and cellular background. Further research is necessary to broaden our understanding of these effects. At the same time, expanding the scope of this combined computational and experimental approach to more immune checkpoints will aid in corroborating the immunomodulatory potential of NTP.

## CRediT authorship contribution statement

**Pepijn Heirman:** Writing – review & editing, Writing – original draft, Visualization, Validation, Methodology, Investigation, Funding acquisition, Formal analysis, Data curation, Conceptualization. **Hanne Verswyvel:** Writing – review & editing, Writing – original draft, Visualization, Validation, Methodology, Investigation, Funding acquisition, Formal analysis, Data curation, Conceptualization. **Mauranne Bauwens:** Writing – review & editing, Visualization, Investigation, Formal analysis. **Maksudbek Yusupov:** Writing – review & editing, Methodology, Investigation, Conceptualization. **Jorrit De Waele:** Writing – review & editing. **Abraham Lin:** Writing – review & editing, Supervision, Funding acquisition, Conceptualization. **Evelien Smits:** Writing – review & editing, Supervision, Resources, Project administration, Funding acquisition. **Annemie Bogaerts:** Writing – review & editing, Supervision, Resources, Project administration, Funding acquisition.

## Declaration of competing interest

The authors declare that they have no known competing financial interests or personal relationships that could have appeared to influence the work reported in this paper.

## Data Availability

Data will be made available on request.
