# Supplementary material for: Effect of plasma-induced oxidation on NK cell immune checkpoint ligands: A computational-experimental approach
Source: Redox Biol. 2024 Oct 1;77:103381. doi: 10.1016/j.redox.2024.103381 (PMC11663777; doi:10.1016/j.redox.2024.103381)
Supplement: Multimedia component 1 [file mmc1.docx]

**Supplementary Data**


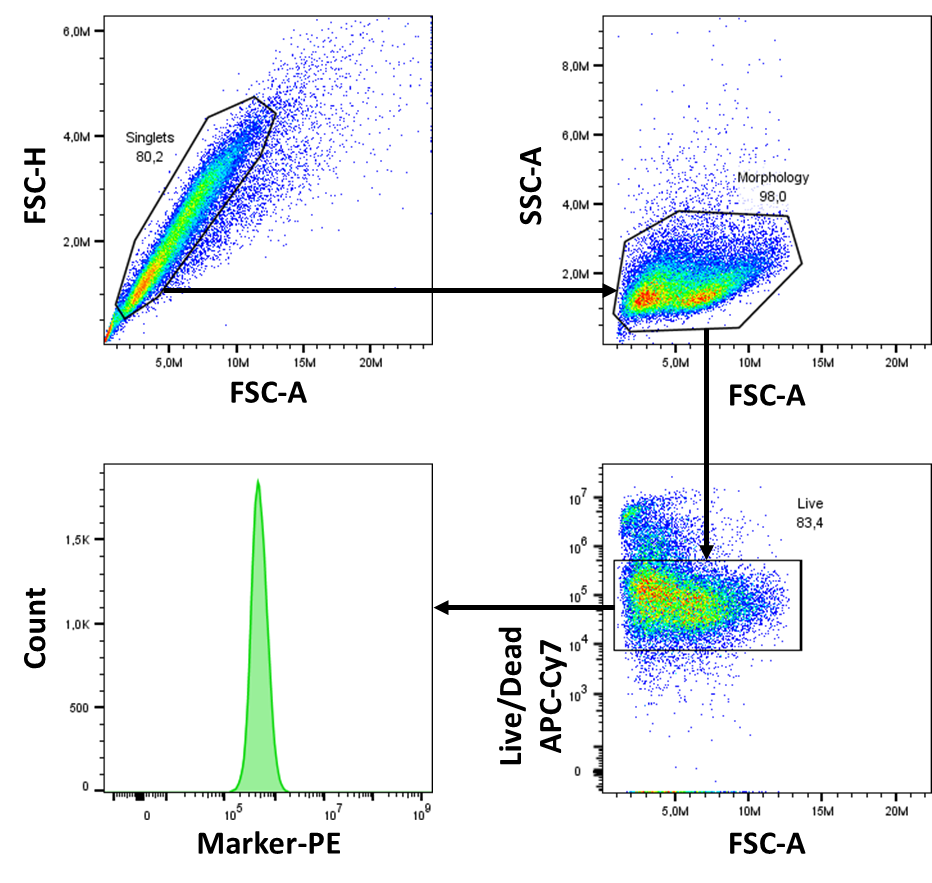


**Supplementary Figure 1: Flow cytometry plots of untreated Cal27 HNSCC cells, representing the gating strategy of all treatment conditions and cell lines throughout the experiment.** HNSCC cell lines (Cal27, SCC61, and SCC22B) were stained for the target (anti-HLA-C, anti-HLA-E, anti-CD155, anti-CD122, anti-CD73, or anti-MICA/B) in the PE channel, and counterstained with a live-dead stain (LIVE/DEAD™ Fixable Near-IR, APC-Cy7). A single cell population was obtained by doublet removal in the forward scatter height and area (FSC-H vs FSC-A) plot, whereafter singlets were gated on morphology in the forward and side scatter area (FSC-A vs SCC-A) plot. Expression of the target-PE was determined in the APC-Cy7^-^ live cell population.


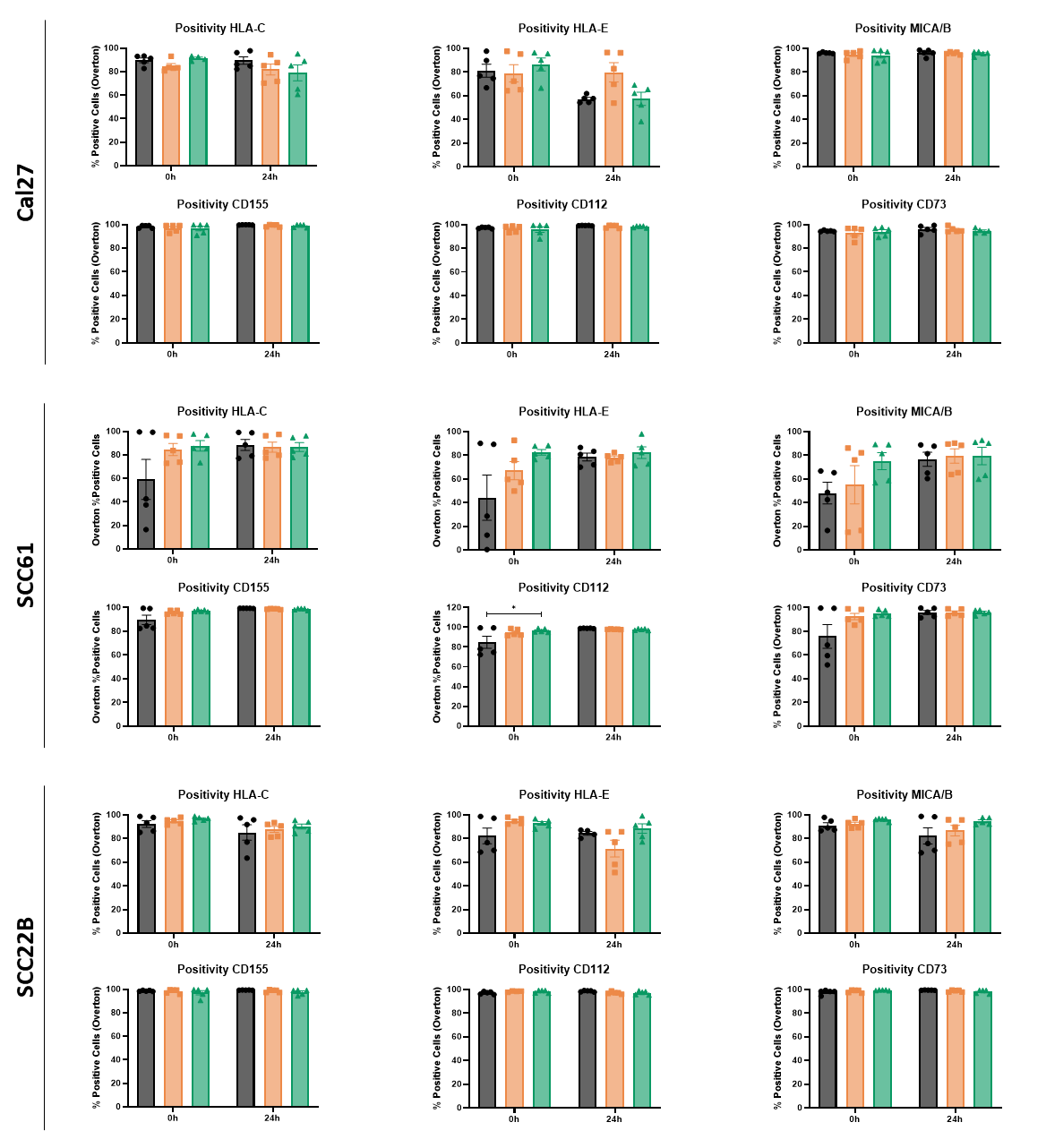


**Supplementary Figure 2: NTP treatment does not affect the percentage of tumor cells positive for the evaluated immune checkpoints.** Quantification of the percentage positive cells after exposure to different NTP intensities, compared to FMO control. Results are depicted as Overton %, immediate (0h) and one day (24h) post-treatment for all 3 HNSCC cell lines. Data are represented as mean ± SEM, with individual values shown (n = 5). Statistical significance between untreated cells and the treated conditions was determined using the generalized linear mixed model with post hoc Dunnett’s test (* p ≤ 0.05). Outliers were calculated with the Grubbs’ Test.

**Supplementary Figure 3: NTP application results in minor changes in CD47 expression in several HNSCC cell lines.** Quantification of the amount of CD47 expression after exposure to different regimes of NTP. Results are depicted as mean fluorescence intensity minus FMO control, normalized to untreated controls (normalized (Norm) ΔMFI), immediate (0h) and one day (24h) post-treatment for all 3 HNSCC cell lines. Data are represented as mean ± SEM, with individual values shown (n = 5). Statistical significance between untreated cells and the treated conditions was determined using the generalized linear mixed model with post hoc Dunnett’s test (* p ≤ 0.05). Outliers were calculated with the Grubbs’ Test.
